# Opposing Effects of Low Molecular Weight Heparins on the Release of Inflammatory Cytokines from Peripheral Blood Mononuclear Cells of Asthmatics

**DOI:** 10.1371/journal.pone.0118798

**Published:** 2015-03-04

**Authors:** Madhur D. Shastri, Niall Stewart, Mathew Eapen, Gregory M. Peterson, Syed Tabish R. Zaidi, Nuri Gueven, Sukhwinder Singh Sohal, Rahul P. Patel

**Affiliations:** 1 School of Medicine, Division of Pharmacy, Faculty of Health, University of Tasmania, Hobart, Tasmania, Australia; 2 Breathe Well Centre of Research Excellence for Chronic Respiratory Disease and Lung Ageing, School of Medicine, Faculty of Health, University of Tasmania, Hobart, Tasmania, Australia; University of Leuven, Rega Institute, BELGIUM

## Abstract

**Background:**

T-cell-mediated inflammatory cytokines, such as interleukin (IL)-4, IL-5, IL-13 and tumor necrosis factor-alpha (TNF-α), play an important role in the initiation and progression of inflammatory airways diseases. Low-molecular-weight heparins (LMWHs), widely used anticoagulants, possess anti-inflammatory properties making them potential treatment options for inflammatory diseases, including asthma. In the current study, we investigated the modulating effects of two LMWHs (enoxaparin and dalteparin) on the release of cytokines from stimulated peripheral blood mononuclear cells (PBMCs) of asthmatic subjects to identify the specific components responsible for the effects.

**Methods:**

PBMCs from asthmatic subjects (consist of ~75% of T-cells) were isolated from blood taken from ten asthmatic subjects. The PBMCs were pre-treated in the presence or absence of different concentrations of LMWHs, and were then stimulated by phytohaemagglutinin for the release of IL-4, IL-5, IL-13 and TNF-α. LMWHs were completely or selectively desulfated and their anticoagulant effect, as well as the ability to modulate cytokine release, was determined. LMWHs were chromatographically fractionated and each fraction was tested for molecular weight determination along with an assessment of anticoagulant potency and effect on cytokine release.

**Results:**

Enoxaparin inhibited cytokine release by more than 48%, whereas dalteparin increased their release by more than 25%. The observed anti-inflammatory effects of enoxaparin were independent of their anticoagulant activities. Smaller fractions, in particular dp4 (four saccharide units), were responsible for the inhibitory effect of enoxaparin. Whereas, the larger fractions, in particular dp22 (twenty two saccharide units), were associated with the stimulatory effect of dalteparin.

**Conclusion:**

Enoxaparin and dalteparin demonstrated opposing effects on inflammatory markers. These observed effects could be due to the presence of structurally different components in the two LMWHs arising from different methods of depolymerisation. This study provides a platform for further studies investigating the usefulness of enoxaparin in various inflammatory diseases.

## Introduction

Unfractionated heparin (UFH), a member of the glycosaminoglycan family, is a complex heterogeneous mixture of polysulfated chains comprised of alternating disaccharide residues of D-glucosamine and uronic acid residues linked by 1→4 glycosidic bonds [1]. The well-known biological role of UFH is its ability to influence blood coagulation and it has been extensively used in clinical practice as an anticoagulant [2]. In recent years, UFH has largely been replaced by low-molecular-weight heparins (LMWHs) for the treatment and prophylaxis of deep vein thrombosis because of more favourable pharmacokinetic properties and with a reduced rate of side effects [3,4]. LMWHs are modified derivatives of UFH obtained by either chemical or enzymatic depolymerisation of UFH [5]. The key structural unit of heparins (UFH and LMWHs) responsible for their anticoagulant activity consists of three D-glucosamine and two uronic acid residues (known as a pentasaccharide sequence). This pentasaccharide sequence binds to the serine protease inhibitor anti-thrombin III and induces conformational changes within the structure of anti-thrombin, thereby accelerating its interaction and subsequent inhibition of thrombin and/or factor Xa of the coagulation cascade [6]. However, not all fragments, also known as oligosaccharides, within heparins contain an anti-thrombin specific pentasaccharide sequence. For example, merely 20–50% of the oligosaccharides of UFH contain the specific anti-thrombin binding domain and the bulk of the oligosaccharides are composed of relatively non-specific sequences, also known as non-anticoagulant oligosaccharides [7,8].

It is now recognised that besides the well-recognised anticoagulant effect, heparins also exhibit a broad spectrum of anti-inflammatory and immune-modulating properties [9–13]. The anti-inflammatory effect of heparins is thought to be due to their ability to alter the activity of a wide range of proteins, such as adhesion molecules, growth factors, cytotoxic mediators and tissue-destructive enzymes [14]. Clinical studies have reported the successful use of heparins for the treatment of chronic obstructive pulmonary disease [10], cancer [11], ulcerative colitis (UC) [12] and lichen planus [13]. The anti-angiogenesis effect in cancer has been shown to be mediated through suppression of tumor vascular endothelial growth factor expression [15]. Anti-UC properties are thought to be exhibited by inhibiting the recruitment of neutrophils, as well as healing of ulcerated mucosa by restoring the high-affinity receptor binding of fibroblast growth factor [16]. Similarly, the therapeutic effectiveness of heparins in lichen planus is thought to be mediated by competitive inhibition of an important component of the extracellular matrix, known as heparinase [17].

A number of clinical studies have also reported the beneficial effects of heparins in asthma [9,18–21]. However, the mechanisms behind these effects are not well understood and there are plausible inflammatory pathways that remain to be explored. Despite being a complex disorder, the aetiology and pathophysiology of asthma is relatively well understood. Cytokines play a pivotal role in orchestrating the inflammation and structural changes of the airways in asthma. Among several types of important inflammatory mediators, T-cell mediated cytokines are known to be the key drivers of respiratory inflammation [22]. During early respiratory inflammation, the activated *naïve* T-helper cells release several inflammatory mediators, including IL-4, IL-5 and IL-13 [23]. IL-4 and IL-5 rapidly attract and prime eosinophils and mast cells. These cells, when activated, release high quantities of IL-5 and tumor necrosis factor (TNF)-α [24]. TNF-α sustains lung inflammatory responses by increasing the accumulation and activation of neutrophils and eosinophils in the airways. Their activation triggers the release of cytotoxic products, further damaging the airways [25]. IL-13 is associated with airway hyper-responsiveness, mucus production and structural changes in the airways called airway remodelling [26]. Also, IL-13 is known to play a key role in corticosteroid-resistant asthma by diminishing binding affinity between corticosteroids and their receptor ligands present on the surface of immune cells [27]. This is clinically significant as it is estimated that up to 10% of asthmatic patients have difficult-to-treat asthma that is often resistant to first line treatment with inhaled corticosteroids [28]. Given the high prevalence of asthma [29], 10% of asthmatic patients represent a significant number. Therefore, the development of potential therapeutic agents targeting difficult-to-treat asthma is highly desirable.

Because of the ability of highly negatively charged heparins to interact with a wide range of biological molecules, we and others have postulated that the possible anti-asthmatic activity of heparins is caused by their inhibitory effects on the release of important inflammatory mediators involved in the pathogenesis of asthma [30–32]. Therefore, in the current *ex-vivo* study we investigated the ability of two widely used LMWHs (enoxaparin and dalteparin) to modulate the T-cell mediated release of IL-4, IL-5, IL-13 and TNF-α in asthmatic subjects, with the aim of identifying the specific oligosaccharide(s) responsible for the anti-inflammatory activity of the parent LMWH.

## Materials and Methods

### Reagents

Enoxaparin (Clexane, 20 mg/0.2 mL; 2,000 IU/0.2 mL) was obtained from Aventis Pharma Ltd. (NSW, Australia). Dalteparin (Fragmin, 16 mg/0.2 mL; 2500 IU/0.2 mL) was purchased from Pfizer Inc. (NSW, Australia). Fondaparinux (Arixtra, 2.5 mg/0.5mL) was purchased from GlaxoSmithKline (Victoria, Australia). RPMI-1640 cell culture medium, Histopaque, antibiotics (penicillin G and streptomycin), phytohaemagglutinin (PHA), ethanol, hydrogen peroxide, sodium hydroxide, acetic acid, potassium hydroxide, sodium sulfate and lactate dehydrogenase (LDH) activity assay kits were purchased from Sigma-Aldrich (Castle Hill, NSW, Australia). Fetal bovine serum was obtained from Invitrogen (Grand Island, NY, USA). ELISA kits for IL-4, IL-5, IL-13 and TNF-α were purchased from Mabtech Australia Pty. Ltd. (Victoria, Australia). Fluroaldehyde assay reagent was purchased from Pierce (Rockford, IL, USA). The anti-factor Xa (AFXa) assay kit was purchased from American Diagnostica (Stamford, CT, USA). Ultrafiltration disk membranes were purchased from Millipore (NSW, Australia). Heparin-derived unsaturated oligosaccharide standards were purchased from V-LABS (Covington, LA, USA).

### Study Sample

A total of 10 healthy (mean age: 34.4 years; range: 28–48 years, 8 males, 2 females) and 10 asthmatic (mean age: 45.6 years; range: 39–51 years, 4 males, 6 females) subjects were recruited from the Medical Science Precinct, University of Tasmania, Australia. The healthy volunteers were not suffering from any acute or chronic diseases and the asthmatic subjects were suffering from no other diseases apart from mild asthma. The asthmatic subjects had not consumed systemic or inhaled corticosteroids or any other immunomodulatory medications to control their asthma in the 2 months before the blood samples were drawn. No information of either the use of other medications or forced expiratory volume (FEV1) was obtained from the recruited participants.

### Ethics Statement

All volunteers were recruited by invitation and the research protocol was approved by the Health and Medical Human Research Ethics Committee (Tasmania, Australia) Network (Approval number: H0013117). Written informed consent for the collection of blood samples was obtained from the recruited volunteers.

### Isolation of Peripheral Blood Mononuclear Cells (PBMCs)

Whole blood (80 mL) from each volunteer was collected and mixed with an equal volume of incomplete RPMI-1640 culture medium to reduce the density of blood, allowing efficient separation of PBMCs from other cells. Aliquots of blood (30 mL) were layered over 20 mL of Histopaque (a density gradient cell separation medium). Following centrifugation at 200g for 30 minutes at 20°C (Eppendorf; Model: 5810R), PBMCs were aspirated from the Histopaque/aqueous interface and centrifuged at 700g for 10 minutes. Cells were washed twice with serum-free medium and resuspended in complete medium [RPMI-1640 supplemented with 2.0 mM L-glutamine, 10% fetal bovine serum and antibiotics (penicillin G and streptomycin)].

### Preparation of Stock Solutions

Stock solutions of enoxaparin, dalteparin and fondaparinux at 1 mg/mL were prepared in RPMI-1640 medium and filtered sterile through 0.2 μm pore size syringe filters (Pall Life Sciences, Victoria, Australia). Similarly, stock solution of PHA (2.5 mg/mL in RPMI-1640 medium; stored at-20°C) was prepared. The stock solutions of LMWHs were serially diluted with RPMI-1640 medium to obtain concentrations over the range of 5 to 1000 μg/mL. Likewise, PHA was diluted with RPMI-140 medium to obtain a concentration of 10 μg/mL.

### Stimulation and LMWHs Treatment of PBMCs

PBMCs were cultured in 24-well cell culture plates at a concentration of 1 × 10^6^ cells/mL/well and stimulated with 10 μg/mL of PHA (T-cell specific stimulant) in the presence of either RPMI-1640 medium (negative control), different concentrations (5, 10, 20, 50, 80 and 100 μg/mL) of enoxaparin or dalteparin, or 5 to 1000 μg/mL of fondaparinux. After 72 hours of incubation (37°C, humidified 5% CO_2_ atmosphere), PBMC cultures were centrifuged, and supernatants were removed and analysed for the levels of released cytokines (IL-4, IL-5, IL-13 and TNF-α) using enzyme-linked immunosorbent assay (ELISA).

### ELISA

Each high protein binding 96-well ELISA plate was prepared as per manufacturer’s recommendations. Briefly, plates were coated with 100 μL/well of capture antibody (diluted in ELISA diluent) and incubated overnight at 4–8°C. The plates were washed twice with wash buffer (200 μL/well); blocked by addition of blocking buffer (PBS with 0.05% Tween 20 containing 0.1% bovine serum albumin) and incubated for 1 hour at room temperature before washing again for 5 times. Stock solutions of cytokine standards were prepared in ELISA diluent. PBMC supernatants and cytokine standards (100 μL/well) were added to plates, incubated for 2 hours and washed 5 times. Next, 100 μL/well of biotinylated detection antibody, at a concentration of 1 μg/mL, was added to each plate and washed 5 times after incubating for 1 hour. Further, 100 μL/well of standard horseradish peroxidase conjugated streptavidin (a commonly used enzyme to modify substrate resulting in colour development) was added to each plate and incubated for 1 hour. Plates were again washed for five times and 3,3′,5,5′-tetramethylbenzidine as a chromogenic substrate solution (100 μL/well) was added. Plates were then allowed to stand in dark for 20–30 minutes and the reaction was quenched using 50 μL/well of stop solution (1N hydrochloric acid). Measurement of the optical density was performed using a plate reader (Spectra Max M2 microplate reader, Sunnyvale, CA) at 450 nm. Each PBMC treatments were performed in triplicates and supernatants of each treatment was analysed in duplicate.

### PBMC Viability and Cytotoxicity Assay

The effect of LMWHs on viability of cells after 72 hours of incubation was assessed using two methods. Firstly, a trypan blue exclusion test was performed on cells and their viability was determined by counting the unstained cells (cells which did not take up trypan blue) using a haemocytometer, as described previously [33]. Secondly, the activity of LDH in culture supernatant was tested to investigate the cytotoxic effect of LMWHs using a LDH *in-vitro* toxicology assay, as described before [34]. The LDH assay kit was prepared as per the manufacturer’s instructions. Briefly, PBMC culture supernatants were centrifuged at 250g for 4 minutes. An aliquot containing 50 μL of either blank (complete medium), control (PBMCs only), cells treated with PHA alone or with PHA in the presence of 100 μg/mL enoxaparin or dalteparin was mixed with 100 μL of solution containing LDH assay mixture (LDH substrate, LDH dye and LDH cofactor). The mixture was then covered with aluminium foil and incubated at room temperature for 20–30 minutes for colour development and the reaction was terminated by the addition of 1 N hydrochloric acid (15 μL). The absorbance was measured spectrophotometrically using a plate reader (Spectra Max M2 microplate reader, Sunnyvale, CA) at a wavelength of 490 nm. Each sample was prepared and analysed in triplicate. Lastly, cell proliferation was carried out by counting the total number of cells 72 hours after PBMC treatments.

### Desulfation of LMWHs

**Complete desulfation of LMWHs**. A solution containing 5 mg/100 μL of enoxaparin and dalteparin was subjected to acid hydrolysis for complete removal of sulfate groups, as described previously [35]. Briefly, 1.5 mL of nitric acid was added to each sample in a capped glass vial and the solution was heated at 80°C overnight before adding 0.3 mL of hydrogen peroxide. The temperature was further raised to 110°C for the following 6 hours. An experimentally determined volume of 1 M sodium hydroxide was used to neutralise the mixture containing nitric acid, hydrogen peroxide and enoxaparin or dalteparin. The neutralised solution was diluted with 8 mL of Milli-Q water and 400 μL of this solution was further diluted to 8 mL.

**N-desulfation of LMWHs**. A solution containing 5 mg/100 μL of enoxaparin or dalteparin was incubated at 50°C for 30 minutes in the presence of tetrahydrofuran (650 μL) and Milli-Q water (50 μL) for partial *N*-desulfation, as described previously [36] with minor modifications. The mixture was neutralised using 0.1 M sodium hydroxide. The resulting product was evaporated to dryness and precipitated by the addition of anhydrous methanol (80% *v/v*) followed by centrifugation at 3000 rpm for 10 minutes. The supernatant was carefully discarded and samples were kept at 4°C overnight. Any remaining traces of methanol were removed using a miVac DNA centrifugal concentrator (Genevac Ltd, Suffolk, UK) and the precipitants were dissolved in 5 mL Milli-Q water.

**Selective 2-O and 3-O-desulfation of LMWHs**. Selective 2-*O* and 3-*O*-desulfation was performed using a previously described method [36]. Briefly, a solution containing 5 mg/100 μL of enoxaparin or dalteparin was dissolved in 0.1 M sodium hydroxide (200 μL). The solution was then frozen and lyophilised to dryness. The residues were dissolved in Milli-Q water (0.5 mL) and the pH was adjusted to 8 by the addition of acetic acid solution. The solution was dialysed against water for 2 days and lyophilised to obtain 2-*O*, 3-*O*-desulfated enoxaparin and dalteparin fragments.

Free sulfate content, free amino groups and anticoagulant activity of completely and selectively desulfated enoxaparin samples was investigated, along with their effects on the PHA-induced release of cytokines from activated PBMCs of the asthmatic subjects.

### Ion Chromatography Analysis of Free Sulfate Content

Free sulfate content of completely and selectively desulfated LMWHs was determined using a previously developed ion chromatography (IC) method [37]. Samples were injected into a Dionex DX-120 instrument (Sunnyvale, CA, USA) consisting of a GP50 gradient pump and AS50 auto sampler. Mobile phases were composed of potassium hydroxide (KOH) and Milli-Q water. Hydroxide eluent gradients were prepared through mixing of KOH solution and Milli-Q water online using a Dionex EluGen II KOH cartridge. A Dionex IonPac AS11 column was used to detect the sulfate content with the optimised KOH eluent gradient from 1 mM to 15 mM over a period of 15 minutes. A total flow rate of 1.0 mL/minute and an injection volume of 25 μL was maintained. Conductivity detection in suppression mode was carried out using a CD25 conductivity detector. Instrument control and data acquisition were performed using Chromeleon software. Sodium sulfate containing 0–20 μg/mL of sulfate was used to prepare the standard curves. For comparison, the presence of free sulfate content in intact LMWHs (before desulfation) was also investigated after ultra-filtration of enoxaparin/dalteparin at 15000 rpm for 10 minutes. Each sample was prepared in triplicate and analysed in duplicate.

### Analysis of Free Amino Groups

The determination of free amino groups in selectively desulfated LMWHs was determined using a sensitive fluoraldehyde-based assay, as described previously [38]. Briefly, selectively desulfated samples (20 μL) were mixed with deionised water (180 μL) before the addition of fluoraldehyde assay reagent (1 mL). The solution was mixed well and fluorescence was measured at 455 nm after excitation at 360 nm using a fluorescence spectrophotometer (model 1605–10S, Perkin-Elmer, Tokyo, Japan). Standard curves (0 to 600 μg/mL) were prepared using glycine.

### Analysis of Anticoagulant Activity

The potentiating effect of intact or desulfated LMWHs on anti-thrombin III inhibition of activated factor Xa was determined as previously described [38]. Briefly, a low-volume microtitre plate anti-FXa assay was performed by incubating (3 minutes, 37°C) a solution containing anti-thrombin III, FXa and intact or desulfated LMWH samples, followed by addition of FXa substrate and further incubation for 10 minutes. The reaction was quenched using glacial acetic acid and the intensity of developed colour was spectrophotometrically measured at 405 nm (Multiskan Go, SkanIt software, Thermo Fisher Scientific).

### Fractionation and Collection of LMWH Fractions

Fractionation of enoxaparin and dalteparin was performed using a previously developed high-performance size-exclusion chromatography (HP-SEC) method [38] with some modifications. A high-performance liquid chromatography (HPLC) system consisting of a Prostar 230 solvent delivery module, a Prostar 335 DAD detector and a Prostar 410 autosampler (Varian, Melbourne, Australia) was utilised. Data acquisition and instrument control were carried out using Star Chromatography Workstation software. The analyses were performed by injecting 10 mg/mL of enoxaparin or dalteparin using a 200 μL sample loop and a 70 μL sample injection volume. UV detection was monitored at 232 nm. Isocratic elution of LMWHs was performed on a Superdex 10/300 GL (300×10 mm) size-exclusion column (GE Healthcare Bio-Sciences, Uppsala, Sweden) using 0.3 M sodium chloride eluent at a flow rate of 0.2 mL/minutes. After each HP-SEC injection (n = 20), 8 fractions of enoxaparin and 9 fractions of dalteparin were collected. The collected fractions were concentrated on a miVac DNA centrifugal concentrator at 40°C and subsequently desalted using PD MidiTrap G-10 columns (GE Healthcare Life Sciences, Uppsala, Sweden). Each fraction was filter sterilized using 0.2 μm pore size syringe filters (Pall Life Sciences, Victoria, Australia) and stored at 4°C until further use. Fractions were analysed in triplicate for their effects on PHA-induced release of cytokines, as well as for their anticoagulant activity as described above.

### Statistical Analysis

Data are presented as mean ± standard deviation (SD) or as percentage change in the release of cytokines following treatments (enoxaparin, dalteparin, desulfated enoxaparin/dalteparin or HPSEC-derived fractions of enoxaparin/dalteparin) compared to the control samples. Statistical analysis was performed using GraphPad Prism (version 6, GraphPad Software Inc, CA, USA), and significance was evaluated using independent sample or paired Student’s *t*-test, and one way analysis of variance (ANOVA), where applicable, followed by Dunnett’s multiple comparison test. A *p*-value of <0.05 was considered statistically significant.

## Results and Discussion

### Release of Cytokines from Stimulated PBMCs

**Fig 1:**
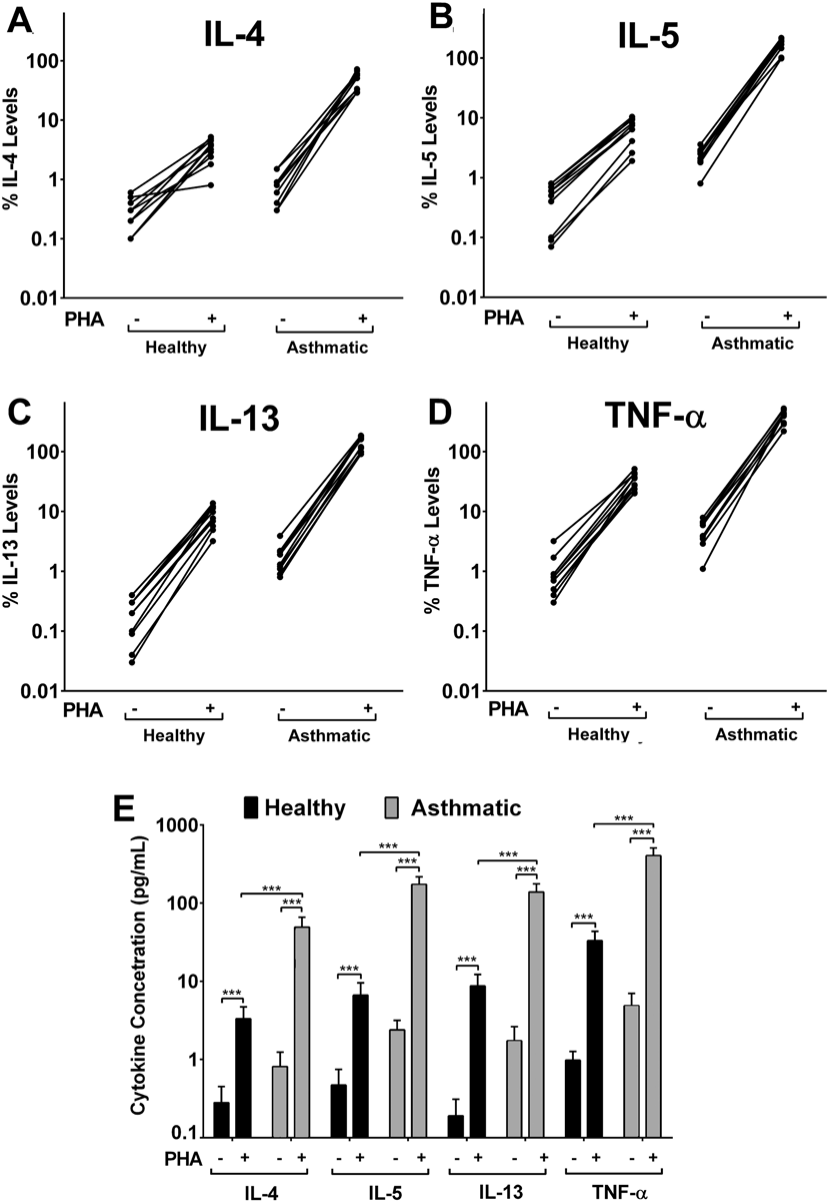
*Ex-vivo* cytokine release. PHA-induced release of IL-4, IL-5, IL-13 and TNF-α in PBMC culture supernatants of healthy (n = 10) and mild asthmatic (n = 10) subjects **(A to D)**. Error bars are omitted for reasons of clarity. **(E)** Summary of the results from A to D. Each sample was analysed in triplicate. Error bars indicate the standard deviation. ***p<0.001 versus PHA-stimulated control.

The levels of the four cytokines (pg/mL) measured 72 hours after PHA-induced stimulation of PBMCs from healthy and asthmatic volunteers are shown in Fig. 1. The calibration curves used for the measurement of cytokines were generated using seven recommended concentrations of respective cytokine standards. The linearity, estimated by correlation coefficient (r^2^), was greater than 0.964 for each of the cytokines. As expected, the levels of tested cytokines from PBMCs of asthmatic subjects were significantly higher than those released from healthy volunteers. It is known that the relative concentrations of the two main types of T-helper cells (Th1 and Th2) are different in patients with asthma compared to healthy individuals. In the healthy population, Th1/Th2 balance is maintained by producing the required Th1 or Th2 cells to establish the normal immune tolerance [39]. In patients suffering from allergic disorders, including asthma, the Th1/Th2 balance becomes abnormal and it shifts abruptly towards Th2 cells [39]. The greater population of Th2 cells upon activation release various cytokines, including IL-4, IL-5, IL-13 and TNF-α.

### Effect of LMWHs on Cytokine Release

**Fig 2:**
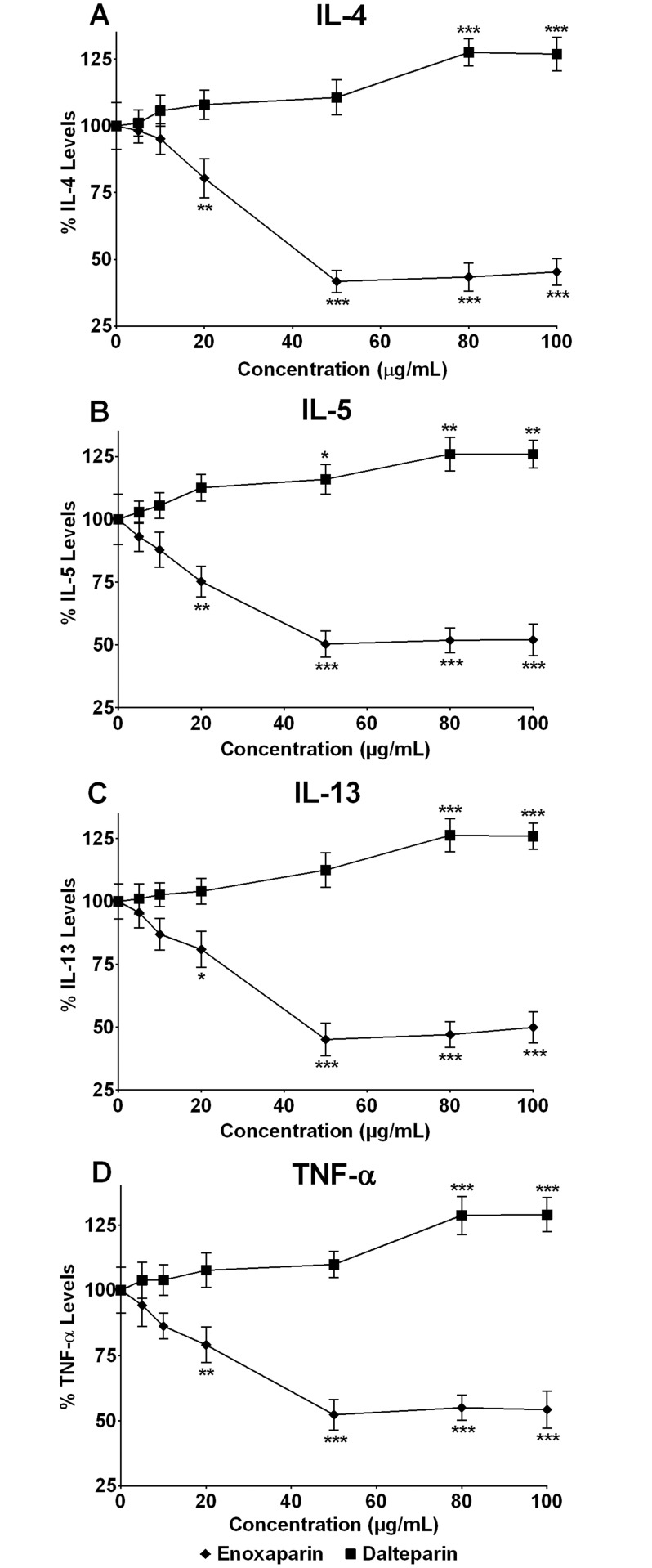
Concentration-dependent effect of LMWHs on cytokine release. Effect of different concentrations of enoxaparin and dalteparin on cytokine levels [IL-4 **(A)**, IL-5 **(B)**, IL-13 **(C)** and TNF- α **(D)**] following PHA-induced *ex-vivo* stimulation of PBMCs from asthmatic subjects (n = 10). Data is presented as percentage (mean ± SD) of the maximal observed cytokine concentrations. *p<0.05, **p<0.01 and ***p<0.001 versus PHA-stimulated control.

To investigate the effects of LMWHs on the release of cytokines, enoxaparin or dalteparin was added to the cells prior to the addition of PHA. The percentage inhibition of cytokine release in the presence or absence of enoxaparin and dalteparin is shown in Fig. 2. The inhibitory effect of enoxaparin was found to be concentration-dependent and its maximum effect was observed at 50 μg/mL (Fig. 2A to 2D). The release of IL-4, IL-5, IL-13 and TNF-α was inhibited by more than 58%, 50%, 55% and 48%, respectively, in the presence of 50 μg/mL of enoxaparin. On the other hand, dalteparin failed to supress the release of tested cytokines and, on the contrary, the release of IL-4, IL-5, IL-13 and TNF-α was increased by more than 25% in the presence of 80 or 100 μg/mL of dalteparin (Fig. 2A to 2D).

Enoxaparin and dalteparin are prepared by different depolymerisation processes [5]. Enoxaparin (an average molecular weight of 4500Da) is prepared by chemical β-eliminative cleavage of benzyl ester of UFH and dalteparin (an average molecular weight 6000Da) is prepared by nitrous acid induced deaminative cleavage of UFH. Therefore, their oligosaccharides have different sequences as well as terminal reducing and non-reducing ends. Compared to dalteparin, enoxaparin is more heterogeneous in nature and is mainly composed of oligosaccharides ranging from dp2 (two saccharide units) to dp16 (16 saccharide units) [40]. On the other hand, the smallest oligosaccharide unit found in dalteparin is dp8 and it mainly contains oligosaccharides longer than dp12 [40]. In line with our results, other studies have also shown different responses of various cells and molecules involved in the process of inflammation to LMWHs. It has been proposed that different effects might result from the presence of different proportions of small and large oligosaccharide chains within various LMWHs [41,42]. Based on this, it can be postulated that the suppression of cytokine release could be due to shorter oligosaccharide chains (≤dp8), prevalent in enoxaparin, whereas an increase in cytokine release might be due to longer oligosaccharides (>dp8), which are common in dalteparin. It has been shown that Th1-type cytokines such as interferon-γ and IL-12 play an important role in controlling immune and allergic responses in asthma [43]. For example, interferon-γ may counteract Th2 mediated immune responses by 1) minimising the development of Th2 cells, 2) increasing the production of IL-12, 3) inducing apoptosis of eosinophil and 4) preventing immunoglobulin switch in B cells. The current study investigated the effects of LMWHs on the release of Th2- but not Th1-type cytokines. Therefore, future studies should be focused on determining the effects of LMWHs on the level of Th1-type cytokines as well.

### Effect of LMWHs on Viability and Proliferation of PBMCs

**Fig 3:**
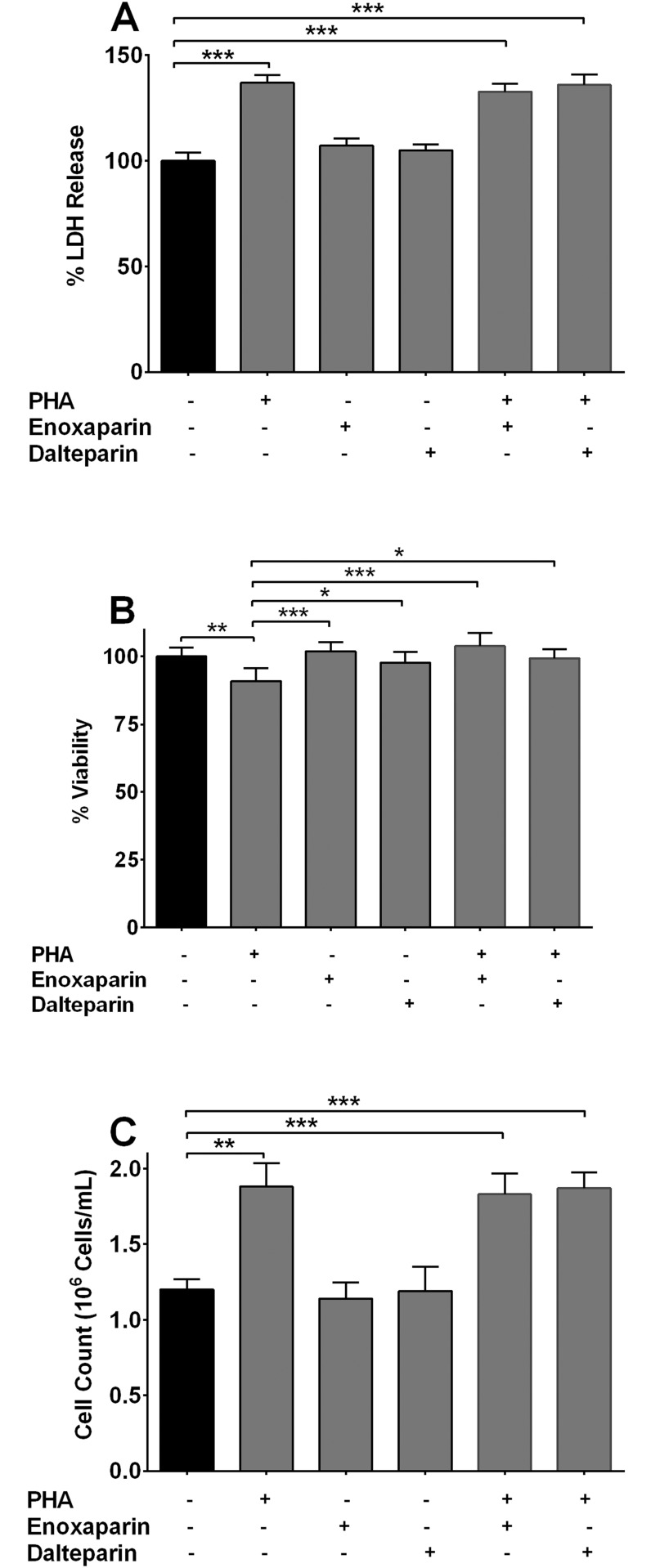
Effect of LMWHs on cellular viability. **(A)** LDH activity of PBMC supernatants from asthmatic subjects (n = 5) expressed as the percentage of maximum LDH release following 72 hours of incubation in the presence of either PHA, enoxaparin/dalteparin alone or enoxaparin/dalteparin co-incubated with PHA. Error bars indicate standard deviation. ***p<0.001 compared to unstimulated cells only control. **(B)** Effect of enoxaparin and dalteparin on the viability of PBMCs obtained from asthmatic subjects (n = 5) after pre-treatment of cells with either PHA, enoxaparin/dalteparin alone or enoxaparin/dalteparin co-incubated with PHA. Viability was measured using trypan blue dye exclusion test 72 hours after incubation with drugs and is presented as the percentage of viable cells. Error bars indicate standard deviation. *p<0.05, **p<0.01 and ***p<0.001 compared to either unstimulated cells only or PHA-stimulated control. **(C)** The effects of enoxaparin and dalteparin on PHA-induced proliferation of PBMCs obtained from asthmatic subjects (n = 5) measured 72 hours after incubation with enoxaparin/dalteparin alone or enoxaparin/dalteparin co-incubated with PHA. Error bars indicate standard deviation. **p<0.01 and ***p<0.001 compared to either unstimulated cells only or PHA-stimulated control.

Different types of heparins have shown to possess pro- or anti-proliferative effects [44,45]. Therefore, it was important to determine whether or not the observed effect above on cytokine production was due to either enoxaparin-induced cell toxicity or dalteparin-induced cell proliferation. The cell viability was assessed in the presence of PHA alone or in combination with PHA and enoxaparin/dalteparin, by detecting the release of LDH (Fig. 3A). Neither enoxaparin nor dalteparin increased the release of LDH, whereas PHA, as described before, induced cellular toxicity [46,47]. PHA-induced toxicity was not affected when PHA-stimulated cells were co-incubated with enoxaparin or dalteparin. Cellular viability in the presence or absence of enoxaparin and dalteparin is shown in Fig. 3B. Unlike PHA, enoxaparin or dalteparin did not produce signs of cellular cytotoxicity. Cell proliferation in the presence or absence of enoxaparin and dalteparin is shown in Fig. 3C. The proliferation of PBMCs was found to be significantly increased in the presence of PHA, whereas enoxaparin and dalteparin showed no effect on cell proliferation. These results indicate that modulation of cytokine release in the presence of tested LMWHs was not related to their cytotoxicity or changes in cellular proliferation.

### Effect of Desulfated LMWHs on Cytokine Release

The anticoagulant activity of LMWHs is strongly influenced by the degree of sulfation and the distribution of sulfate groups in their oligosaccharide chains [1]. To investigate whether the observed effect of enoxaparin and dalteparin was dependent on their anticoagulant activity, they were subjected to complete or selective desulfation. Free sulfate in intact enoxaparin and dalteparin was determined by IC and was found to be equivalent to 0.42% and 0.36% (w/w), respectively. Total bonded sulfate in enoxaparin and dalteparin after complete desulfation, and allowing for the free sulfate, was found to be 38.7 and 42.3% (w/w), respectively. This value for enoxaparin or dalteparin was similar to the theoretical estimation of sulfate content (40% of enoxaparin and 44% w/w of dalteparin) based on 75% trisulfation of the heparin disaccharide repeating unit from porcine mucosa and an average of one *N*-acetyl group per parent heparin molecule and the type of their reducing ends (2-*N*, 6-*O*-disulfo-D-glucosamine or 1,6-anhydro groups for enoxaparin and 6-*O*-sulpho-2,5-anhydro-D-mannitol for dalteparin).

The effect of completely desulfated enoxaparin and dalteparin on the release of TNF-α (levels of which were found to be highest in PBMC supernatants) was examined. Unlike intact LMWHs, completely desulfated molecules did not inhibit or increase the release of cytokines, indicating the importance of sulfate groups for retaining the observed activity of LMWHs. The key structural unit of heparins that confers anticoagulant activity is an oligosaccharide sequence consisting of three D-glucosamine and two uronic acid residues. The anticoagulant activity of heparins has been shown to be dependent on *N*- and *O*-sulfate groups present in the oligosaccharide sequence. Elimination of the *N*-sulfate groups results in decreased anticoagulant activity, but the elimination of 3-*O*-sulfate group of the central D-glucosamine results in the loss of anticoagulant activity by approximately 20,000 times [1].

**Fig 4:**
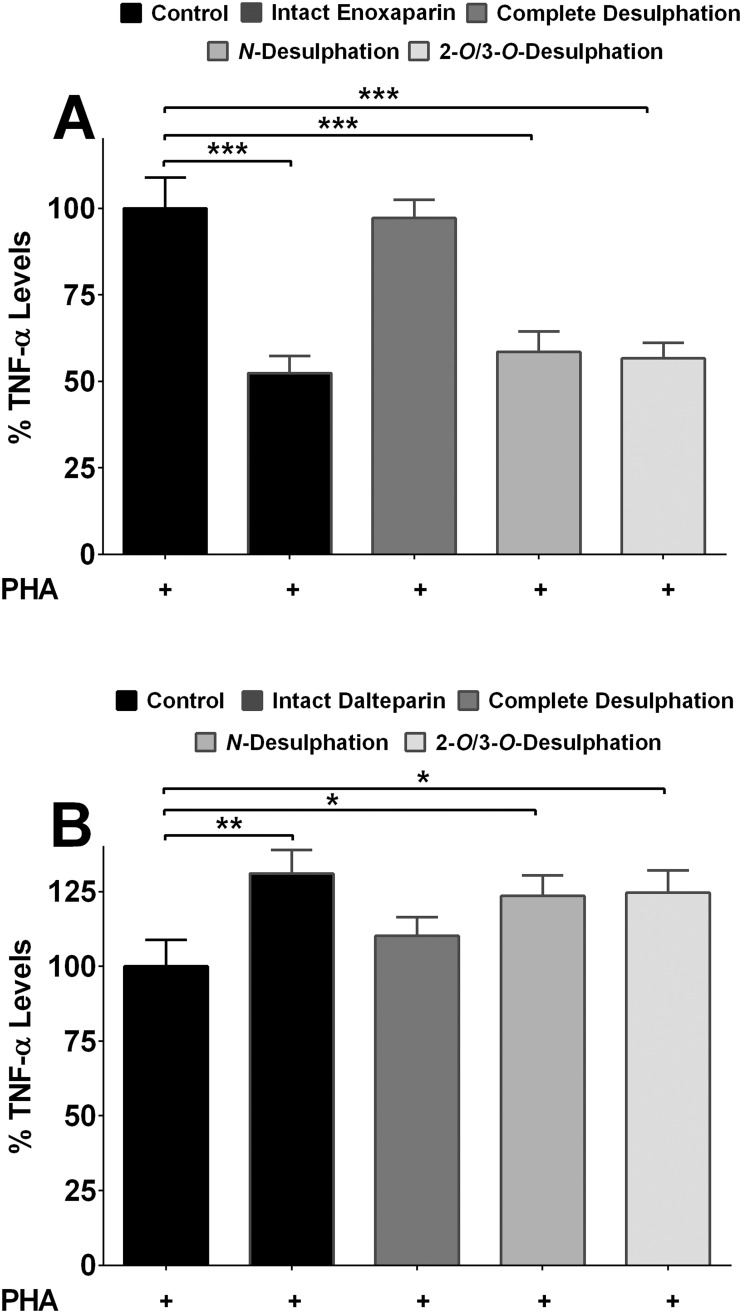
Effect of de-sulfated LMWHs on cytokine release. Suppression of TNF-α release by completely desulfated, *N*-desulfated or 2-*O*/3-*O*-desulfated fragments of enoxaparin **(A)** or dalteparin **(B)** after PHA-induced stimulation of PBMCs from asthmatic subjects (n = 5). Error bars indicate standard deviation. *p<0.05, **p<0.01 and ***p<0.001 compared to PHA-stimulated control.

To determine the role of *N*- and 2-*O*/3-*O* sulfate groups in any pro- or anti-inflammatory effect, enoxaparin and dalteparin were selectively desulfated. *N*-desulfation and 2-*O*/3-*O* desulfation was confirmed by commonly used fluoraldehyde assay and IC, respectively (data not shown). It was found that enoxaparin and dalteparin retained their activity in the absence of *N*- and 2-*O*/3-*O* sulfate groups (Fig. 4A and 4B) indicating these groups are not important for their observed effects. Therefore, the anti- or pro-inflammatory effects of LMWHs were independent of their anticoagulant activity.

Clinical studies have indicated that LMWHs can potentially be used in human inflammatory disorders [30,48]. However, they possess both anticoagulant and non-anticoagulant oligosaccharides. Therefore, their potential use is hampered by the risk of bleeding in conditions other than where anticoagulation is required [49]. To investigate whether the anti-inflammatory effect of enoxaparin is separable from its anticoagulant effect, enoxaparin was separated and the oligosaccharide(s) responsible for its anti-inflammatory effect was identified before determining the anticoagulant effect of the identified oligosaccharide(s). Dalteparin was also separated to identify the oligosaccharide(s) responsible for its pro-inflammatory effect.

### Separation and Identification of Oligosaccharides

**Fig 5:**
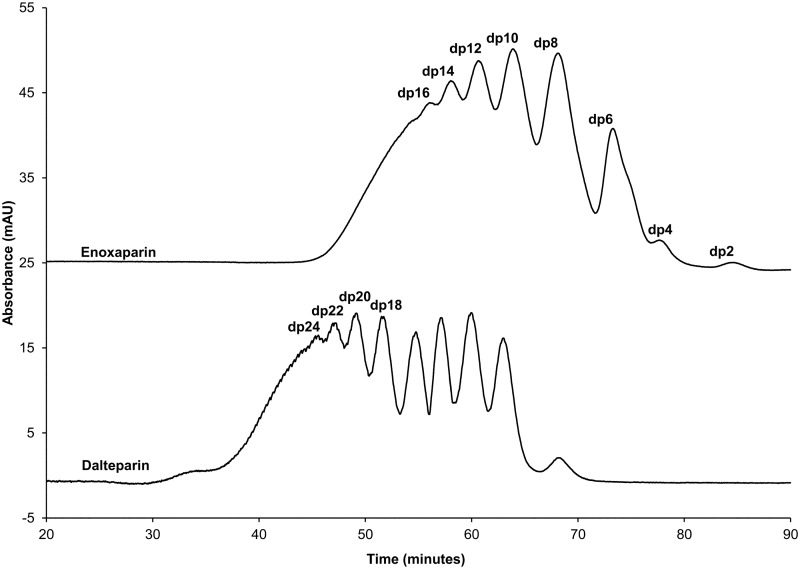
HPSEC analysis of enoxaparin and dalteparin. The HP-SEC methodology is described in the experimental section. Approximate saccharide composition of the separated peaks of enoxaparin and dalteparin is shown; dp2- two saccharide units to dp24- twenty four saccharide units.

The HP-SEC chromatograms of enoxaparin and dalteparin are presented in Fig. 5. As expected, different chromatographic profiles of enoxaparin and dalteparin were observed. The saccharide composition of each HP-SEC separated peak of enoxaparin and dalteparin has been reported before [40]. Enoxaparin was separated into 8 different peaks representing dp2 to dp16, whereas dalteparin was separated into 9 different peaks representing dp8 to dp24 (Fig. 5).

Due to structural complexity and high negative charge, LMWHs cannot be effectively separated without prior depolymerisation. Therefore, different techniques, such as reversed-phase high-performance liquid chromatography [50] and capillary electrophoresis [51], have been used for the separation of depolymerised heparin derivatives. However, elevated temperature or freeze drying processes used during depolymerisation can result in structural modifications of the oligosaccharides and therefore certain biological effects of intact LMWHs are lost or altered after depolymerisation process [38]. Hence, enoxaparin and dalteparin in this study were separated using the HP-SEC method. This technique can separate oligosaccharides without the need for chemical or enzymatic depolymerisation of the parent molecule. Although this technique is most widely used for the identification of saccharide composition of LMWHs, it has its own specific limitations. For example, structurally different oligosaccharides having the same or similar saccharide composition cannot be separated using this technique. Therefore, it is possible that the HP-SEC separated fractions of LMWHs (e.g. dp4 of enoxaparin) are composed of structurally different molecules having the similar saccharide composition.

### Effect of Identified Oligosaccharides on Cytokine Release

**Fig 6:**
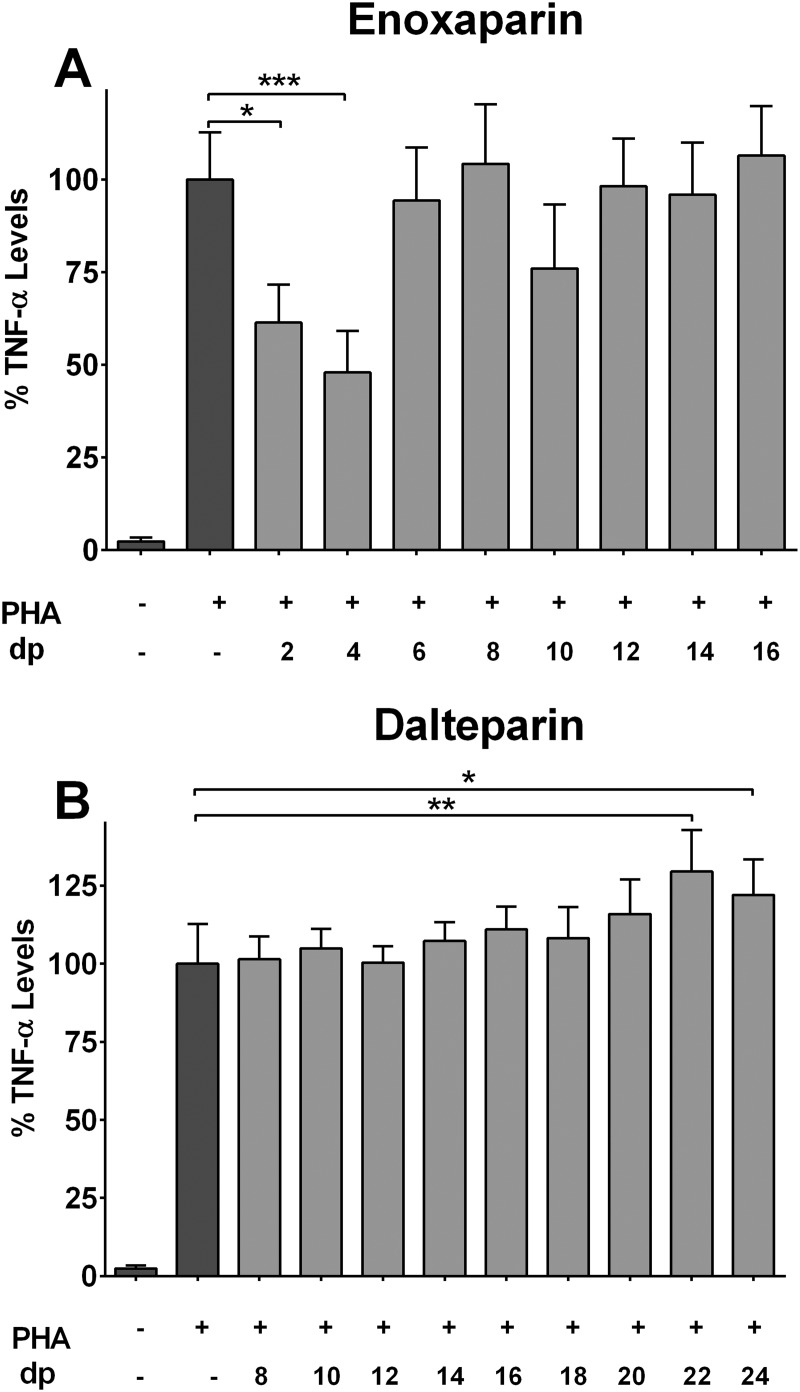
Effect of HPSEC fractions of LMWHs on cytokine release. Suppression of TNF-α release by eight HP-SEC derived saccharides (dp2 to dp16) of enoxaparin **(A)** or nine HP-SEC derived saccharides (dp8 to dp24) of dalteparin **(B)** after PHA-induced stimulation of PBMCs from asthmatic subjects (n = 5). Error bars indicate standard deviation. *p<0.05, **p<0.01 and ***p<0.001 versus PHA-stimulated control.

Separated fractions of enoxaparin and dalteparin were collected and then re-analysed by HP-SEC to confirm their saccharide composition and purified using desalted columns. The ability of each desalted fraction to modulate the release of TNF-α is shown in Fig. 6. The tested concentration of each fraction was based on its actual concentration, calculated using the peak area, present in 50 μg/mL of enoxaparin or 80 μg/mL of dalteparin. The release of TNF-α was significantly increased by 15%, 29% and 21% in the presence of dp20, dp22 and dp24 saccharides of dalteparin, respectively. On the other hand, dp8 to dp18 did not significantly modulate cytokine release, indicating the oligosaccharides larger than dp18 of dalteparin have pro-inflammatory activity (Fig. 6B). Fondaparinux, a synthetic LMWH, was used to determine whether the observed pro-inflammatory effect of dalteparin fractions were independent to their anticoagulant effects. Fondaparinux is composed of only pentasaccharide sequence responsible for the anticoagulant activity of LMWHs. It, at tested concentrations (5 to 1000 μg/mL), failed to inhibit or enhance the levels of IL-4, IL-5, IL-13 and TNF-α (data not shown) suggesting the observed effect of dalteparin molecules were not dependent to their anticoagulant activity.

Several autoimmune diseases are associated with down regulation of T-cell receptor signalling pathways, resulting in dysfunction of T-cells [52]. This may have important consequences such as insufficient response to various types of infections [53]. The therapeutic potential of dalteparin in such medical conditions could potentially be explored owing to its stimulatory effect on T-cells. HP-SEC separated dp2 and dp4 of enoxaparin inhibited the release of TNF-α by 39 and 52%, respectively (Fig. 6A). Dp6 showed some activity but it was not statistically different from the control samples and the fractions larger than dp6 did not show a significant inhibitory effect (Fig. 6A) suggesting the shorter oligosaccharides are responsible for the anti-inflammatory effect of enoxaparin. This finding is important because a minimum of five saccharide chain length with specific sulfation pattern is required for the anticoagulant activity of any type of LMWH. One of the major concerns against the development of LMWHs as an anti-asthmatic agent is the bleeding risk associated with its use. Disaccharide (dp2) or tetrasaccharide (dp4) chains are not sufficiently long enough to bind AT-III and therefore do not exhibit anticoagulant activity (which was confirmed by previously described low-volume microtitre plate anticoagulant assays). Hence, these fractions of enoxaparin would potentially minimise the risk of bleeding associated with intact enoxaparin and could be investigated further as potential therapeutic targets for the treatment of inflammatory diseases including asthma.

In conclusion, the current study provides a solid platform for further experimental and clinical studies. Future research should be designed focusing on: i) further separation of dalteparin’s dp22 and enoxaparin’s dp4 molecules, with the aim of identification and structural elucidation of the saccharide moieties responsible for the pro- or anti-inflammatory activity of the parent LMWHs; ii) identification of underlying cellular and molecular mechanisms by which dp22 of dalteparin and dp4 of enoxaparin modulate the T-cell induced release of pro-inflammatory cytokines; and iii) pre-clinical and clinical studies confirming the observed non-anticoagulant activities of the identified enoxaparin oligosaccharides.
